# Medical Malpractice in the Management of Angioedema: A Multidisciplinary Westlaw Analysis

**DOI:** 10.1002/wjo2.70119

**Published:** 2026-06-11

**Authors:** Emma De Ravin, Neel R. Sangal, Daniel Uralov, Karthik Rajasekaran

**Affiliations:** ^1^ Department of Otolaryngology – Head and Neck Surgery Thomas Jefferson University Philadelphia Pennsylvania USA; ^2^ Department of Otorhinolaryngology – Head and Neck Surgery University of Pennsylvania Philadelphia Pennsylvania USA; ^3^ Division of Otolaryngology Children's Hospital of Philadelphia Philadelphia Pennsylvania USA; ^4^ Leonard Davis Institute of Health Economics University of Pennsylvania 3641 Locust Walk Philadelphia Pennsylvania USA

**Keywords:** angioedema, litigation, malpractice, otolaryngology, Westlaw database

## Abstract

**Background:**

The management of acute angioedema is challenging and involves providers in multiple specialties. Timing of evaluation and intervention is imperative and requires effective communication between these groups, as treatment delays and improper management can lead to airway compromise and death. We aim to identify factors that contribute to malpractice litigation in the management of angioedema.

**Materials and Methods:**

The Westlaw comprehensive legal database was queried for angioedema malpractice cases from 1993 to 2022. Angioedema misdiagnoses were excluded. Root cause analysis was conducted and cases were classified by defendant specialty, claimed liabilities, complication, verdict, and damage amount awarded.

**Results:**

Nine cases of angioedema malpractice were identified and met inclusion criteria. Eight cases (89%) resulted in patient death due to cardiorespiratory compromise secondary to loss of the airway, with one case resulting in long‐term brain damage. Root cause analysis revealed 67% of cases involved failure to establish a timely airway, 56% delayed treatment, 33% failure to diagnose, and 33% improper treatment. Forty‐five percent of the involved practitioners were emergency physicians; primary care and anesthesia were each implicated in one case. Five (56%) cases ruled in favor of the defense. Average damage amount was $3,794,000. In each case, there was significant delay in the involvement of Otolaryngologists for airway evaluation.

**Conclusions:**

Angioedema is a challenging disease for which management requires active monitoring and coordinating of care between several medical teams. This study identifies late involvement of airway experts in patient management and risk stratification as a risk factor for litigation and poor outcomes.

## Summary

- Medical malpractice litigation places a large burden on American physicians and healthcare systems. To date, no study has analyzed malpractice litigation in the management of angioedema.
- The two most common alleged liabilities are failure to establish an airway and treatment delay. Delays in diagnosis and appropriate airway evaluation were found in all four cases resulting in payout.
- Ultimately, standardized management algorithms and early involvement of airway experts (e.g., otolaryngologists) may help prevent medical malpractice litigation in these high acuity cases.

## 1 Introduction

Medical malpractice rates have steadily declined over the last two decades, with a 30% decrease in malpractice payment reports filed with the National Practitioner Data Bank between 2011 and 2021 [1, 2]. Despite this trend, among surgeons and physicians in other high‐litigation‐risk specialties, malpractice litigation is a certainty for the majority of providers at some point during the course of their career, with 88% of physicians in high‐risk specialties projected to face their first claim by the age of 45 years [3]. As such, medical malpractice litigation remains a large financial, emotional, and time burden for American physicians and healthcare systems [4].

This burden, and the desire to discourage the practice of defensive medicine, have spurred efforts to mitigate litigation risk and reduce medical errors in order to safeguard both patients and physicians [5]. Primarily, these efforts are centered around tort reform, patient safety initiatives, litigation analysis, and physician education, which are imperative to not only reducing the number of malpractice claims and their associated economic burden, but also improving patient‐centered care and enabling the development of targeted prevention and risk‐management strategies [6, 7, 8].

In otolaryngology, prior studies have examined medical litigation for a variety of head and neck oncologic procedures and pathologies [4, 6, 7, 8, 9, 10, 11, 12, 13] and in the specialty as a whole [4, 8]. However, no study thus far has focused on medical malpractice in the management of acute oropharyngeal angioedema, a potentially life‐threatening condition that, without timely, appropriate treatment, can rapidly lead to airway compromise and death [14]. Angioedema involves acute onset watery, non‐pitting edema of the dermis, subcutaneous, and submucosal tissues of the lips, face, palate, tongue, and larynx [14, 15, 16]. Because of the varying severity in clinical presentation (from edema limited to one facial subsite without airway symptoms to laryngeal edema with airway obstruction) and dynamic progression, angioedema management requires close monitoring and urgent airway evaluation and risk stratification [14, 15, 16]. In this study, we aimed to provide a summary of medical malpractice claims regarding the management of acute angioedema and identify factors that contribute to litigation in these cases.

## 2 Materials and Methods

A retrospective search for all medical malpractice cases involving acute angioedema was conducted via the Westlaw comprehensive legal database from database inception to July, 2022 (WESTLAW, West Publishing Co., St. Paul, MN). The database contains jury verdict reports and formal settlements for all United States federal and state court cases submitted in all 50 states and published by 14 judicial sources; search terms included “malpractice,” “medical malpractice,” and “angioedema.” Cases were reviewed for relevance; angioedema misdiagnoses and cases that did not involve the management of acute angioedema were excluded from analysis. The following data were extracted from each remaining verdict report: case location and court type, date of verdict/settlement, primary injury and liabilities claimed, defendant demographics, defendant specialty, case outcome (defendant vs. plaintiff verdict vs. settlement), and damage amount awarded.

## 3 Results

### 3.1 Database Search Results and Case Demographics

Initial query of the Westlaw legal database identified 17 cases between database initiation and 2022. After deduplication (five cases) and exclusion of angioedema misdiagnoses and cases irrelevant to angioedema management (three cases), nine unique angioedema cases remained.

Of those nine lawsuits, cases were filed in eight states: two in Florida, and one each in Illinois, Massachusetts, Missouri, New Jersey, Pennsylvania, Washington, and West Virginia. There was no temporal trend in litigation, with half of the cases occurring in the former half of the study period (1993–2008) and half occurring in the latter half of the study period (2008–2022). Plaintiffs were 56% female with a mean age of 55 years, while the vast majority of defendants were male (*n* = 9, 82%). Plaintiffs sued hospitals or medical centers in three cases and healthcare providers in seven cases. The most litigated specialty was emergency medicine (*n* = 5, 45%); other litigated specialties included dentistry (*n* = 2, 18%), and one provider each in oral surgery, psychiatry, anesthesiology, and primary care. All case demographic information can be found in Table 1.

**Table 1:** Case characteristics and demographic information.

Case region, *n* (%)	
Northeast	3 (33%)
Southeast	3 (33%)
Midwest	2 (22%)
West	1 (11%)
Court type, *n* (%)	
Circuit court	5 (56%)
District court	1 (11%)
Superior court	3 (33%)
Plaintiff gender, *n* (%)	
Male	4 (44%)
Female	5 (56%)
Plaintiff age, mean ( ± SD)	55 (19.5)
Defendant gender, *n* (%)	
Male	9 (82%)
Female	2 (18%)
Defendant specialty, *n* (%)	
Emergency medicine	5 (45%)
Dentistry	2 (18%)
Oral surgery	1 (9%)
Psychiatry	1 (9%)
Anesthesiology	1 (9%)
Primary care	1 (9%)

### 3.2 Liabilities and Injuries Claimed

Across the nine angioedema cases, eight (89%) resulted in patient death due to cardiorespiratory compromise secondary to loss of the airway. The second most common injury was loss of consortium, which was claimed in two of the cases (22%). Regarding legal allegations, multiple general liabilities were often asserted within a single case. These included death (*n* = 5, 56%), general malpractice (*n* = 4, 44%), physician/health care professional malpractice (*n* = 4, 44%), procedure/treatment malpractice (*n* = 2, 22%), and facility malpractice (*n* = 2, 22%) (Table 2). Because more than one liability could be alleged per case, category totals exceed 100%. Root cause analysis identified 20 specific liabilities, the most common of which being failure to establish an airway (*n* = 6, 67%), delays in treatment (*n* = 5, 56%), misdiagnosis/failure to diagnose (*n* = 3, 33%), and improper treatment (*n* = 3, 33%). Other specific liabilities cited were negligent supervision, failure to communicate, and failure to monitor, each of which was found in one of the nine cases (Table 2).

**Table 2:** Summary of general and specific liabilities claimed.

General liability	*n* (%)
Death	5 (56%)
Malpractice (general)	4 (44%)
Malpractice (physicians and health professionals)	4 (44%)
Malpractice (procedures and treatment)	2 (22%)
Malpractice (facility)	2 (22%)
Specific liability	
Failure to establish airway	6 (67%)
Delayed treatment	5 (56%)
Misdiagnosis/Failure to diagnose	3 (33%)
Improper treatment	3 (33%)
Negligent supervision	1 (11%)
Failure to communicate	1 (11%)
Failure to monitor	1 (11%)

### 3.3 Case Outcomes and Verdicts

The majority of the suits resulted in defense verdicts (*n* = 5, 56%), with the remaining four cases resulting in indemnity payments: one plaintiff verdict (11%), one mixed verdict (11%), and two settlements (22%). Payment amounts ranged from $730,000 (for failure to diagnose angioedema resulting in moderate brain damage and a seizure disorder) to $10,200,000 (for a failure of physician‐physician communication regarding pre‐operative clearance in a patient with a known history of angioedema, resulting in patient death), with an average payment of $3,794,000. Patient death was the primary injury in three of the four payout cases, and delays in diagnosis and appropriate airway evaluation were found in all four cases.

## 4 Discussion

In this cross‐sectional retrospective cohort study, we queried the Westlaw legal database to identify all medical malpractice cases in the management of angioedema between 1993 and 2022. We were then able to characterize the malpractice environment in angioedema care and identify factors that contribute to litigation in these cases. We identified nine unique cases meeting inclusion criteria, among which the vast majority (89%) involved patient death due to loss of the airway. Defendants were most commonly male and emergency medicine physicians. Root cause analysis identified 20 alleged liabilities, the two most common of which being failure to establish an airway and treatment delay. Defendants prevailed in five of the nine cases; the remaining cases resulted in payouts, with an average award of $3,794,000 (range: $730,000–10,200,000). Delays in diagnosis and appropriate airway evaluation were found in all four cases resulting in payout.

Despite declining national malpractice rates on the whole [1, 2], litigation remains a certainty for the majority of physicians in high‐risk specialties at some point in their career [3]. In otolaryngology specifically, more than 50% of surgeons report facing at least one malpractice claim, with more than two claims per otolaryngologist on average [10]. Otolaryngologists work in a high‐stakes area vital to daily function and quality of life—an anatomically complex territory prone to high‐acuity situations that necessitate emergent intervention—allowing for greater understanding of litigation, and making physician education all the more critical [4].

Though litigation analysis has been conducted for sinonasal disease [12], laryngeal cancer [6], and meningitis [9], this study is the first of its kind to examine litigation in acute angioedema management. Angioedema is characterized by acute onset watery subcutaneous or submucosal edema and arises from two different etiologies: hereditary (C1 inhibitor deficiency) and acquired (angiotensin‐converting enzyme [ACE] inhibitors and non‐steroidal anti‐inflammatory drugs [NSAIDs], allergic reactions, and idiopathic causes) [14, 15, 17, 18]. When found in the head and neck, angioedema can involve a variety of subsites in the oropharynx and upper respiratory tract (e.g., face, lips, tongue, floor of mouth, pharynx, larynx), the involvement of which dictate the extent of airway involvement and risk of life‐threatening airway obstruction [14, 15, 19]. Due to the diversity in etiologies and therapeutic agents, the involvement of a variety of healthcare providers, and the risk for significant morbidity and potential mortality, angioedema management is highly complex. As a consequence, it also poses a potential source of malpractice litigation and an important gap in litigation analysis for otolaryngologists and other related healthcare providers [20].

In the present study, we demonstrated a low incidence of malpractice litigation in angioedema management, with only nine lawsuits identified between 1993 and 2022. Importantly, this result likely underrepresents the malpractice claims brought against healthcare providers, as the Westlaw database is limited to cases that make it all the way through the legal pipeline to state or federal court, and does not assess the cases that are settled out of court prior to that point [8]. We also demonstrated no temporal or geographic trends in angioedema litigation, with an equal distribution of cases is the former and latter halves of the time period analyzed. While prior studies have demonstrated significantly higher litigation rates in the Northeast [4, 10, 21, 22], we demonstrated a fairly even distribution of suits between the Northeast (*n* = 3, 33%), Southeast (*n* = 3, 33%), and Midwest (*n* = 2, 22%), with one case (11%) on the West coast, which may be explained by the small sample size in this study.

Of note, understanding the epidemiology of angioedema provides important context for interpreting the geographic distribution of malpractice litigation observed in this study. Angioedema accounts for an estimated 80,000–112,000 emergency department visits annually in the United States, with drug‐induced etiologies, particularly ACE inhibitor‐associated angioedema, representing a substantial proportion of cases [23, 24, 25, 26]. In contrast, hereditary angioedema is rare, affecting approximately 1 in 50,000 individuals [27]. Regional differences may reflect variation in medication exposure, demographic characteristics, and comorbidity burden rather than true geographic clustering of disease. Given the widespread use of ACE inhibitors nationwide, the relatively even geographic distribution of malpractice cases identified in our cohort likely reflects broad population exposure rather than meaningful regional variation in disease prevalence.

Examination of the defendants shows that across nine cases, healthcare providers from six individual specialties were named as defendants. Although multiple specialties (allergy and immunology, otorhinolaryngology, etc.) undergo significant education and training for the management of acute angioedema, we found that emergency medicine physicians were most likely to face malpractice litigation related to angioedema, accounting for 45% of the suits identified. This is perhaps unsurprising, given that primary care and emergency department physicians are most likely to interface with angioedema patients [28]. However, the diversity of specialties that face angioedema litigation reflects the broad array of healthcare providers that may encounter angioedema in their clinical practice, thus necessitating effective provider education in patient management and risk stratification, and in thorough communication among multidisciplinary teams, for effective care of these patients.

Root cause analysis identified “failure to establish an airway,” “delayed treatment,” and “misdiagnosis/failure to diagnose” as the most common sources of malpractice in angioedema management. This provides the impetus and potential for significant improvements in angioedema training and care delivery. One mechanism for such training is via high‐fidelity simulation models, which have been demonstrated to improve trainee clinical knowledge and enable targeted skills acquisition [29]. By expanding existing angioedema educational programs [30] and extending training models to emergency medicine physicians, we will allow for earlier recognition and prompt, appropriate treatment for patients where they most commonly present for care, thus potentially decreasing adverse events and litigation related to angioedema management.

In our analysis, 56% of cases ruled in favor of the defendant, which is congruent with defense verdict rates established in the literature of around 60% [4]. Our study also highlights that trial verdicts appear to be more financially punitive than a settlement. Of the cases that resulted in indemnity payments, the two cases resulting in settlements had lower payouts than the plaintiff or mixed verdicts, on average, though this difference did not reach statistical significance ($902,500 vs. $6,685,500; *p* = 0.241). In the single plaintiff verdict, a patient was administered lisinopril for hypertension and experienced acute onset angioedema. Despite clinical signs of airway compromise (globus sensation, swollen tongue, difficulty breathing), the defendant failed to provide standard of care treatment and delayed sending the patient to the emergency department. Later, when the patient was sent to the emergency department, they failed multiple intubation attempts and ultimately died secondary to cardiopulmonary arrest. Patient death occurred in three of the four payout cases, and delays in diagnosis and appropriate airway evaluation were found in all four cases.

This analysis also highlights opportunities for improved patient care via the implementation of established protocols, algorithms, and clinical practice guidelines for the management of acute angioedema [31, 32, 33, 34, 35, 36, 37, 38]. Any patient presenting with possible angioedema should trigger rapid evaluation from a comprehensive team, which should include an emergency physician, otolaryngologist, pharmacist, and anesthesiologist. Once an angioedema diagnosis is confirmed, the etiology/cause (known drug or food allergies, existing hereditary or acquired angioedema, ACE inhibitor use, family history, etc.) should be identified and avoided when possible. Patients presenting with odynophagia, hoarseness, voice change, dyspnea, or other upper airway complaints should undergo evaluation via flexible fiberoptic nasopharyngolaryngoscopy (NPL) [31, 33, 39]. Further, because of the potential for airway compromise, an airway plan and time frame for monitoring progress must be established. Finally, for any patients with laryngeal edema, observation in a setting with continuous pulse oximetry and close monitoring in a setting like the intensive care unit (ICU) should be encouraged [39].

### 4.1 Limitations

This study is not without its limitations. As discussed previously, though the Westlaw legal research platform is a large, comprehensive database that references [14] judicial sources for its content, there are inherent limitations to analyzing only one database. The Westlaw database (and other similar resources) exclusively identify cases with jury verdict reports, thus nine presented cases do not represent all malpractice allegations regarding angioedema care during the time period and are instead limited to litigation that both made it to state or federal court (and were thus included in the public record, vs*.* being settled beforehand) and reached a final verdict within the study period. Other limitations are related to variability in data collection and information provided by the database, which may have skewed the findings that we presented, given our small cohort. That being said, only one missing entry was noted across all variables collected, thus we concluded that this did not significantly impact the conclusions that we made. Future studies that incorporate all allegations from the Physicians Insurers Association of America (PIAA) data‐sharing report would better enable the evaluation of overall litigation incidence and trends in angioedema care.

## 5 Conclusions

The complexity of angioedema management and the risk for significant morbidity and potential mortality place physicians at risk for medical malpractice. In this study, we characterize angioedema medical malpractice litigation from 1993 to 2022 and identify factors associated with litigation in these cases. Overall, nine malpractice cases were identified over a 29‐year period, with eight of those suits involving cases that resulted in patient death. We identified a variety of healthcare providers implicated in lawsuits related to angioedema management, with the most commonly involved specialty being emergency medicine. For angioedema‐related claims, the most common alleged injuries were failure to establish an airway and delayed treatment. Ultimately, targeted interventions including risk stratification and continuing medical education via high‐fidelity simulation models, will be essential to reducing adverse outcomes and future malpractice litigation in acute angioedema. Future studies should implement standardized management algorithms and integrate risk management strategies to help prevent medical malpractice litigation in these high acuity cases.

## Author Contributions

**Emma De Ravin:** conceptualization, data curation, formal analysis, writing – original draft, writing – review and editing. **Neel R. Sangal:** conceptualization, data curation, formal analysis, writing – review and editing. **Daniel Uralov:** writing – review and editing. **Karthik Rajasekaran:** conceptualization, investigation, project supervision, writing – review and editing.

## Funding

The authors have nothing to report.

## Disclosure

The authors have nothing to report.

## Ethics Statement

This study was performed in compliance with the Helsinki Declaration.

## Conflicts of Interest

Professor Karthik Rajasekaran is a member of World Journal of Otorhinolaryngology – Head & Neck Surgery (WJOHNS) editorial board and is not involved in the peer review process of this article. The remaining authors declare no conflicts of interest.

## Data Availability

The data that support the findings of this study are available from the corresponding author upon reasonable request.
